# Supplementary material for: Distinct risk groups with different healthcare barriers and acute care use exist in the U.S. population with chronic liver disease
Source: PLoS One. 2024 Nov 20;19(11):e0311077. doi: 10.1371/journal.pone.0311077 (PMC11578530; doi:10.1371/journal.pone.0311077)
Supplement: S1 Data — (DOCX) [file pone.0311077.s001.docx]

Data Availability Statement

Submission PONE-D-24-17172R1

Users may download the data directly from [nhis.ipums.org](https://urldefense.com/v3/__http:/nhis.ipums.org__;!!F9wkZZsI-LA!FtTHaeRCLdoxrHTM3BIS4YWTfbGOT0xySPdHWPmJczZO1QGLSafVtQ0bWol5oi4r0FtWB-3oS0h2jDHftw$) following the instructions below:

1. Select “Get Data”
2. Select samples between 2011 and 2017 to add to cart
3. Select variables of interest using the drop-down menu to add to cart. Variables of interest include the following:

| Variable Name | Variable Description |
| --- | --- |
| serial | Sequential Serial Number, Household Record |
| quarter | Sample quarter, household record |
| strata | Stratum for variance estimation |
| psu | Primary sampling unit (PSU) for variance estimation |
| nhishid | NHIS Unique identifier, household |
| hhweight | Household weight, final annual |
| procyear | Processing year |
| region | Region of residence |
| nhispid | NHIS Unique Identifier, person |
| px | Person number of respondent (from NHIS). |
| hhx | Household number (from NHIS) |
| fmx | Family number (from NHIS) |
| pernumhh | Person number within hh (from reformatting) |
| perweight | Final basic annual weight |
| sampweight | Sample Person Weight |
| fweight | Final annual family weight |
| supplwt | Supplemental Person Weight 1 |
| supp2wt | Supplemental Person Weight 2 |
| supp3wt | Supplemental Person Weight 3 |
| supp4wt | Supplemental Person Weight 4 |
| intervwmo | Month of NHIS interview |
| intervwyr | Year of NHIS interview |
| lateinta | Late Sample Adult interviews |
| astatflg | Sample adult flag |
| proxysa | Sample adult needs proxy to answer questions |
| astatqcflag | Quality control flag for sample adult |
| hhrefflag | Household reference person flag |
| saproxyavail | Knowledgeable proxy available for sample adult |
| saproxyrel | Relationship of sample adult proxy |
| interimwt | Weight - Interim Annual |
| age | Age |
| sex | Sex |
| racenew | Self-reported Race (Post-1997 OMB standards) |
| hispeth | Hispanic ethnicity |
| famsize | Number of persons in family |
| educrec2 | Educational attainment recode, intervalled |
| educrec1 | Educational attainment recode, nonintervalled |
| educ | Educational attainment |
| empstatwkyr | Work status: Last week, past 12 months |
| pooryn | Above or below poverty threshold |
| health | Health status |
| eryrno | Number times in ER/ED in past 12 months |
| usualpl | Has usual place for medical care |
| delayappt | Delayed care because couldn’t get an appointment soon |
| delayhrs | Delayed care because doctor’s office not open |
| delayphone | Delayed care because couldn’t get through by phone |
| delaytrans | Delayed care because lacked transportation |
| delaywait | Delayed care because wait too long in doctor’s office |
| ybarcare | Needed but couldn’t afford medical care, past 12 months |
| ybarmeds | Needed but couldn’t afford prescription medicines, past 12 months |
| ybarfollow | Needed but couldn’t afford follow-up care, past 12 months |
| ybarspecl | Needed but couldn’t afford specialist, past 12 months |
| trubfindryr | Had trouble finding general doctor, past 12 months |
| nonewpatyr | Told not accepted as new patient, past 12 months |
| nohctakeyr | Told health care coverage not accepted, past 12 months |
| hinotcove | Health insurance coverage status |
| hipubcove | Has any Medicaid/ other public assistance/ State sponsored plan |
| hipubcov | Has any public insurance |
| hiprivate | Has any private health insurance |
| himcaid | Has Medicaid insurance |
| himcare | Has Medicare insurance |
| angipecev | Ever told had angina pectoris |
| arthglupev | Ever told had arthritis/rheumatoid arthritis/gout/lupus/fibromyalgia |
| asthmaev | Ever told had asthma |
| cancerev | Ever told had cancer |
| cheartdiev | Ever told had coronary heart disease |
| diabeticev | Ever told had diabetes |
| emphysemev | Ever told had emphysema |
| heartattev | Ever told heart attack |
| heartconev | Ever told had heart condition/disease |
| kidneywkyr | Told had weak/ failing kidneys, past 12 months |
| liverchron | Ever had any chronic liver condition |
| liverconyr | Told had liver condition, past 12 months |
| strokev | Ever told had a stroke |
| flany | Has any functional limitation |

1. Create data extract to obtain the data
